# Draft Genome Sequence of *Salmonella enterica* subsp. *enterica* Serovar Gallinarum Biovar Pullorum Strain FCAV198, a Brazilian Chicken Pathogen

**DOI:** 10.1128/genomeA.00028-14

**Published:** 2014-02-20

**Authors:** Diego F. A. Batista, Oliveiro C. Freitas Neto, Laura R. Leite, Alessandro M. Varani, Flavio M. G. Araujo, Anna Salim, Adriana M. Almeida, Simone A. M. Ribeiro, Guilherme C. Oliveira, Paul A. Barrow, Angelo Berchieri Junior

**Affiliations:** a Faculty of Agriculture and Veterinary Sciences, Universidade Estadual Paulista, Jaboticabal (FCAV/Unesp), Sao Paulo, Brazil; b Excellence Center in Bioinformatics (Cebio), Belo Horizonte, Minas Gerais, Brazil; c National Agriculture and Livestock Laboratory (LANAGRO), Campinas, Sao Paulo, Brazil; d School of Veterinary Medicine and Science, University of Nottingham, Loughborough, United Kingdom

## Abstract

*Salmonella enterica* subsp. *enterica* serovar Gallinarum biovar Pullorum is a bird-restricted pathogen which causes pullorum disease. The strain FCAV198 was isolated from a pool of chicken ovaries in Brazil, and its genome may be helpful for studies involving molecular mechanisms related to pathogenesis and other related applications.

## GENOME ANNOUNCEMENT

Pullorum disease (PD), which is caused by *Salmonella enterica* subsp. *enterica* serovar Gallinarum biovar Pullorum, was reported worldwide in 2012, but its occurrence was especially high in Asian countries (http://www.oie.int/wahis_2/public/wahid.php/Diseaseinformation/statusdetail). PD is known as a systemic disease responsible for high mortality in young birds (1). Some of the birds that recover from infection become persistently infected and transmit *Salmonella* biovar Pullorum vertically (2). The bird restriction and aspects of PD pathogenesis, mainly the persistence of infection, are thought to be associated with gene losses (O. C. Freitas Neto, personal communication). The genome decay in *Salmonella* biovar Pullorum, caused by pseudogene accumulation, has already been observed in other *Salmonella enterica* subsp. *enterica* genomes (3–5).

Genetic variability among *Salmonella* biovar Pullorum strains has been demonstrated by use of several different molecular approaches over the past few years (6–8), highlighting the need for more genome sequencing to reveal the core genes. In addition, genomic comparisons among the closely related pathogens *Salmonella* biovar Pullorum and *Salmonella* serovar Gallinarum biovar Gallinarum could be helpful to clarify the genome evolution and molecular basis of their distinct pathogenicity and epidemiology. The *Salmonella* biovar Pullorum strain FCAV198 was isolated in Brazil from a pool of chicken ovaries in 2008. This strain was serotyped and granted by the National Agriculture and Livestock Laboratory (LANAGRO), which is the official laboratory of the Brazilian Ministry of Agriculture, Livestock and Supply and gives support to the surveillance of animal diseases.

The whole genome was sequenced using the SOLiD 4.0 sequencing system, which produced 65 million mate-paired reads of 50 bp, with insert sizes from 1,000 to 2,000 bp. *De novo* assembly was carried out using the CLC Genomics workbench 6.5.1 (CLC bio, Aarhus, Denmark) and generated 128 contigs. The draft genome sequence has a G+C content of 52.2% and a total size of 4,783,588 bp, as expected for *Salmonella* spp. (4, 5). The annotation and metabolic reconstruction were performed using the Prokka pipeline version 1.7 (Prokaryotic Genome Annotation System) and Rapid Annotations using Subsystems Technology (RAST) server (9), respectively. With these methods, 4,652 coding sequences (CDSs; with an average length of 847 bp), 65 tRNA-encoding genes, and 27 rRNA-encoding genes were predicted. It is noteworthy that the last 182,819 bp (from contigs 48 to 127) corresponds to mobile genetic elements, plasmids, and other genomic sequences which had no matches to the closely related *Salmonella* biovar Pullorum strain CDC 1983-67 (NC_022221). Some of these regions may be acquired by lateral gene transfer and encoding of potential biovar-specific traits, such as were observed in other *Enterobacteriaceae* (10, 11).

Experimental studies by *in vivo* infection indicate that the strain FCAV198 is pathogenic for chickens and shows the typical biochemical profile observed in other *Salmonella* biovar Pullorum strains (12). Furthermore, the genetic variability observed among the strains of *Salmonella* biovar Pullorum (6–8) suggests that their genomes are under different evolutive forces. Indeed, the genome sequencing approach of several different biovars could shed light on the biology of *Salmonella* biovar Pullorum and on PD disease, which is still responsible for economic losses to the poultry industry worldwide.

### Nucleotide sequence accession number.

The annotated draft genome sequence was deposited in DDBJ/EMBL/GenBank under accession number AZRG00000000. The version described in this paper is the first version.

## References

[1] Barrow PA Freitas Neto OC 2011 Pullorum disease and fowl typhoid—new thoughts on old diseases: a review. Avian Pathol. 40:1–13. 10.1080/03079457.2010.542575 21331943

[2] Berchieri Junior A Murphy CK Marston K Barrow PA 2001 Observations on the persistence and vertical transmission of *Salmonella enterica* serovars pullorum and gallinarum in chickens: effect of bacterial and host genetic background. Avian Pathol. 30:221–231. 10.1080/03079450120054631 19184904

[3] Feng Y Xu HF Li QH Zhang SY Wang CX Zhu DL Caor FL Li YG Johnston RN Zhou J Liu GR Liu SL 2012 Complete genome sequence of *Salmonella enterica* serovar Pullorum RKS5078. J. Bacteriol. 194:744. 10.1128/JB.06507-11 22247537 PMC3264078

[4] Thomson NR Clayton DJ Windhorst D Vernikos G Davidson S Churcher C Quail MA Stevens M Jones MA Watson M Barron A Layton A Pickard D Kingsley RA Bignell A Clark L Harris B Ormond D Abdellah Z Brooks K Cherevach I Chillingworth T Woodward J Norberczak H Lord A Arrowsmith C Jagels K Moule S Mungall K Sanders M Whitehead S Chabalgoity JA Maskell D Humphrey T Roberts M Barrow PA Dougan G Parkhill J 2008 Comparative genome analysis of *Salmonella* Enteritidis PT4 and *Salmonella* Gallinarum 287/91 provides insights into evolutionary and host adaptation pathways. Genome Res. 18:1624–1637. 10.1101/gr.077404.108 18583645 PMC2556274

[5] Holt KE Thomson NR Wain J Langridge GC Hasan R Bhutta ZA Quail MA Norbertczak H Walker D Simmonds M White B Bason N Mungall K Dougan G Parkhill J 2009 Pseudogene accumulation in the evolutionary histories of *Salmonella enterica* serovars Paratyphi A and Typhi. BMC Genomics 10:36. 10.1186/1471-2164-10-36 19159446 PMC2658671

[6] Li J Smith NH Nelson K Crichton PB Old DC Whittam TS Selander RK 1993 Evolutionary origin and radiation of the avian-adapted non-motile salmonellae. J. Med. Microbiol. 38:129–139. 10.1099/00222615-38-2-129 8429538

[7] Olsen JE Skov MN Christensen JP Bisgaard M 1996 Genomic lineage of *Salmonella enterica* serotype Gallinarum. J. Med. Microbiol. 45:413–418. 10.1099/00222615-45-6-413 8958244

[8] Porwollik S Santiviago CA Cheng P Florea L Jackson S McClelland M 2005 Differences in gene content between *Salmonella enterica* serovar Enteritidis isolates and comparison to closely related serovars Gallinarum and Dublin. J. Bacteriol. 187:6545–6555. 10.1128/JB.187.18.6545-6555.2005 16159788 PMC1236623

[9] Aziz RK Bartels D Best AA DeJongh M Disz T Edwards RA Formsma K Gerdes S Glass EM Kubal M Meyer F Olsen GJ Olson R Osterman AL Overbeek RA McNeil LK Paarmann D Paczian T Parrello B Pusch GD Reich C Stevens R Vassieva O Vonstein V Wilke A Zagnitko O 2008 The RAST server: rapid annotations using subsystems technology. BMC Genomics 9:75. 10.1186/1471-2164-9-75 18261238 PMC2265698

[10] Kingsley RA Msefula CL Thomson NR Kariuki S Holt KE Gordon MA Harris D Clarke L Whitehead S Sangal V Marsh K Achtman M Molyneux ME Cormican M Parkhill J MacLennan CA Heyderman RS Dougan G 2009 Epidemic multiple drug resistant *Salmonella* Typhimurium causing invasive disease in sub-Saharan Africa have a distinct genotype. Genome Res. 19:2279–2287. 10.1101/gr.091017.109 19901036 PMC2792184

[11] Nie H Yang F Zhang X Yang J Chen L Wang J Xiong Z Peng J Sun L Dong J Xue Y Xu X Chen S Yao Z Shen Y Jin Q 2006 Complete genome sequence of *Shigella flexneri* 5b and comparison with *Shigella flexneri* 2a. BMC Genomics 7:173. 10.1186/1471-2164-7-173 16822325 PMC1550401

[12] Crichton PB Old DC 1990 *Salmonellae* of serotypes gallinarum and pullorum grouped by biotyping and fimbrial-gene probing. J. Med. Microbiol. 32:145–152. 10.1099/00222615-32-3-145 1973735

